# Characterization of the complete chloroplast genome of *Actinidia Melanandra* (Actinidiaceae)

**DOI:** 10.1080/23802359.2019.1623129

**Published:** 2019-07-11

**Authors:** Yuemei Zhao, Zhixin Zhao, Weili Tang, Xiaoling Li, Yongping Zhao, Baoyun Gao, Xiaodan Xie, Xiaobin Zhang

**Affiliations:** a College of Biopharmaceutical and Food Engineering, Shangluo University, Shangluo, China;; b College of Health Management, Shangluo University, Shangluo, China

**Keywords:** *Actinidia melanandra*, chloroplast, Illumina sequencing, phylogenetic analysis

## Abstract

The whole chloroplast (cp) genome sequence of *Actinidia melanandra* has been characterized from Illumina pair-end sequencing. The complete cp genome was 156,124 bp in length, containing a large single-copy region (LSC) of 88,006 bp and a small single-copy region (SSC) of 20,332 bp, which were separated by a pair of inverted repeat (IR) regions of 23,893 bp. The genome contained 131 genes, including 84 protein-coding genes, 39 tRNA genes, and 8 ribosomal RNA genes (4 rRNA species). Most genes occur as a single copy, while 17 gene species are duplicated. Phylogenetic analysis revealed that *A. melanandra* is closely related to the species of *A. deliciosa* and *A. chinensis.*

*Actinidia melanandra* is a kind of dioecious climbing species belonging to the family Actinidiaceae. It is an endemic species to China and distributed in broad-leaved forests, mountain forests, or moist places of central and southern China (Flora of China Editorial Committee of Chinese Academy of Sciences 2007). *Actinidia melanandra* can produce clusters of sweet reddish berries which cannot be produced commercially because of its short shelf-life. Research suggests this plant has a good performance in cold resistant abilities and survive at temperatures till −20 °C. In view of this feature, this species has been used for breeding of new cultivars in recent years (Kaya et al. 2016). Therefore, it is fundamental to understand the genomic information of *A. melanandra.* In this study, we assembled and characterized the complete chloroplast genome of *A. melanandra* from Illumina sequencing data.

The fresh leaves of a single individual of A. melanandra were collected from Shangluo (Shaanxi, China; 108°37′E, 33°26′N) and Voucher herbarium specimens (ZA16015) were deposited at the Herbarium of Shangluo University. Genomic DNA was extracted from the fresh leaves using the CTAB method (Doyle 1987). Total DNA was used for the shotgun library construction and the subsequent high-throughput sequencing on the Illumina HiSeq 2500 Sequencing System. In total, 3.1G raw reads were obtained, quality-trimmed and used for the cp genome assembly using MITObim v1.8 (Hahn et al. 2013) with *Actinidia chinensis* (GenBank: NC_026690.1) (Yao et al. 2015) as the initial reference. The genome was visualized and annotated in Geneious version 9.0.2 (Biomatters Ltd., Auckland, New Zealand). The circular plastid genome map was completed using the online program OGDRAW (Lohse et al. 2013). A neighbor-joining (NJ) tree was inferred using MEGA6.0 (Tamura et al. 2013) from alignments created by the MAFFT (Katoh and Standley 2013) using nine other complete chloroplast genomes previously reported in Actinidiaceae. The annotated genomic sequence has been submitted to GenBank with the accession number MK863365.

The circular chloroplast genome of *A. melanandra* was 156,124 bp in size, and comprises a pair of inverted repeat (IR) regions of 23,893 bp each, a large single-copy (LSC) region of 88,006 bp, and a small single-copy (SSC) region of 20,332 bp. The chloroplast genome contained 131 genes including 84 protein-coding genes, 39 tRNA genes, and 8 rRNA genes. In these genes, 16 genes contained one intron and two genes contained two introns. The majority of the gene species are single copy; however, 17 gene species in the IR regions are totally duplicated, including 5 protein-coding genes, 8 tRNA genes, and 4 rRNA genes. Out of these 17 gene species, rps12 are partially located within the IR regions, while all the others completely within the IR regions. The overall GC content of *A. melanandra* chloroplast genome is 37.2%.

Phylogenetic analysis was performed using the neighbor-joining (NJ) method with 1000 bootstrap replicates based on 10 complete chloraplast genome sequences of Actinidiaceae, of which *Clematoclethra scandens* subsp. *hemsleyi* and *Sladenia celastrifolia* were used as outgroup. As shown in the highly resolved NJ phylogenetic tree (Figure 1), all the species of the genus *Actinidia* formed a monophyletic clade with a high resolution value and *A. melanandra* was closely related to *A. deliciosa* and *A. chinensis.*

**Figure 1.**
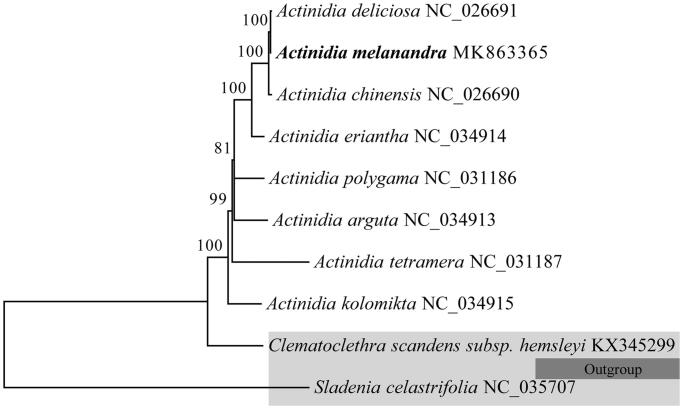
Neighbor-joining (NJ) phylogenetic tree based on 10 complete chloroplast genomes.
